# Antiplatelet Therapy for Stroke Prevention in Atherosclerotic Cardiovascular Disease-Naïve People with Cerebral Small-Vessel Disease: A Retrospective Cohort Study

**DOI:** 10.3390/jcm15145704

**Published:** 2026-07-21

**Authors:** Do-Hyung Kim, Nak Gyeong Ko, Jong-Ho Park

**Affiliations:** 1 Department of Neurology, Samsung Changwon Hospital, Sungkyunkwan University School of Medicine, Changwon 51353, Republic of Korea; 2 Department of Research & Support, Samsung Changwon Hospital, Sungkyunkwan University School of Medicine, Changwon 51353, Republic of Korea; nakgyeong.ko@samsung.com; 3 Department of Neurology, Eunpyeong St. Mary’s Hospital, The Catholic University of Korea, Seoul 03312, Republic of Korea

**Keywords:** cerebral small vessel disease, white matter hyperintensities, stroke, magnetic resonance imaging

## Abstract

**Background/Objective:** White matter hyperintensities (WMH) are frequently observed in adults without atherosclerotic cardiovascular disease (ASCVD), yet antiplatelet agents are often prescribed empirically. We aimed to explore whether antiplatelet treatment is associated with stroke prevention in this specific population. **Methods:** This retrospective analysis used prospectively collected data from adults undergoing regular health examinations (2011–2019). We assessed associations between antiplatelet use and stroke outcomes according to WMH Fazekas (1–3) and cerebral small-vessel disease (cSVD) scores (0–4). The primary outcomes were stroke of any type, ischemic stroke, and hemorrhagic stroke. **Results:** Among 1672 participants (mean age 57.1 ± 8.5 years; 15.6% on antiplatelets), 28 (1.7%) strokes of any type, 26 (1.6%) ischemic strokes, and 2 (0.2%) hemorrhagic strokes occurred during a mean 3.5-year follow-up. The risk of any stroke was higher in those with cSVD scores 3–4 compared to score 0 (adjusted HR 8.45; 95% CI 1.53–46.53). WMH severity was not independently related to increased risk of any outcome events. The relative risk of those with cSVD scores 3–4 for stroke of any type and ischemic stroke was not different across subgroups stratified by antiplatelet use (all *P*_interaction_ > 0.05). Post hoc power analysis showed 0.12 power for both strokes of any type and ischemic strokes. **Conclusions:** While cSVD score may help identify higher stroke risk, our findings suggest that antiplatelet agents might not provide significant additional benefit for stroke prevention in ASCVD-naïve adults. These results should be considered hypothesis-generating, emphasizing the need for careful risk factor management over empirical antiplatelet prescription.

## 1. Introduction

White matter hyperintensities (WMH) are a key imaging biomarker of cerebral small-vessel disease (cSVD) and are frequently observed in older individuals [1]. Since WMH confer an increased risk of stroke, cognitive decline and death [2,3,4], antiplatelet agents are often prescribed in clinical practice for individuals with WMH without atherosclerotic cardiovascular disease (ASCVD), driven by the desire to mitigate the perceived high risk of future stroke and cognitive decline. The clinical benefit of antiplatelet therapy for secondary prevention in patients after clinical stroke is well-established, but its efficacy for the primary stroke prevention in neurologically asymptomatic individuals with WMH remains unknown. Indeed, there have been no robust randomized controlled trials or established guidelines for the management of incidentally found WMH on brain MRI. The ESO (European Stroke Organisation) guideline advises against antiplatelet medications to be prescribed for cSVD per se for primary stroke prevention [5] and the 2024 AHA/ASA (American Heart Association/American Stroke Association) guideline does not recommend antiplatelet agents to reduce the risk of ischemic stroke due to lack of evidence supporting efficacy (Class of Recommendation 2b, Level of Evidence C) [6]. A previous longitudinal study on 83 subjects with silent lacunar infarcts but no prior ASCVD found that low-dose aspirin did not significantly reduce the main outcome events including stroke, TIA, or new silent infarcts compared to the non-treated group (two versus nine events; *p* = 0.10) [7].

The disparity between expert recommendations and off-label clinical practice underscores the need to definitively answer the question about whether long-term antiplatelet therapy provides a net clinical benefit—specifically, reducing the risk of first stroke—in individuals with incidental WMH. With this background, we explored the relationship between WMH and stroke outcomes in association with the antiplatelet effect among individuals free of ASCVD. Additionally, we sought to elucidate the efficacy of antiplatelet agents in association with cSVD burden.

## 2. Materials and Methods

### 2.1. Study Participants

This is a retrospective cohort study that included individuals aged over 20 years who visited the Total Healthcare Center of Samsung Changwon Hospital for regular health examinations from January 2011 to December 2019. There were 3703 participants who underwent brain MRI, or MRI and MRA. In South Korea, self-referred premium health-screening programs are widely prevalent, allowing asymptomatic individuals to voluntarily undergo brain MR imaging as part of a routine check-up, often sponsored by their employers or funded out-of-pocket for proactive health management. Consequently, the primary indication for brain MRI in this cohort was routine health surveillance in neurologically healthy individuals, rather than the evaluation of acute neurovascular events. Excluded from this study were participants (1) whose Fazekas score was 0 (*n* = 1609), (2) with incomplete MR data for analyzing cSVD (*n* = 65), (3) who had a history of ASCVD (*n* = 211), comprehensively assessed via structured self-reported questionnaires, clinician interviews, and institutional electronic medical records encompassing clinically diagnosed stroke, myocardial infarction, angina pectoris, coronary revascularization, or peripheral arterial disease, and (4) who had no information on whether or not they were taking antiplatelet agents (*n* = 146). Thus, a total of 1672 neurologically healthy participants were included in the final analysis (Figure S1).

### 2.2. Data Collection

Data on demographics, clinical characteristics, laboratory findings and brain MRI, and medication information on antiplatelet (e.g., aspirin, clopidogrel, cilostazol, or sarpogrelate), antihypertensive, and antidiabetic agents, and lipid modifiers (statin with/without ezetimibe) were retrieved. To ensure operational efficiency on the day of the health examination, baseline demographics and ASCVD history were pre-screened 1 to 2 weeks prior to the scheduled health examination date using structured questionnaires and institutional EMR reviews. Regular exercise was defined as at least 150 min of moderate-intensity aerobic physical activity per week, or 75 min of vigorous-intensity aerobic physical activity per week [8]. Fasting blood samples were collected in the morning after an overnight fast for over 12 h. The laboratory analyses encompassed plasma glucose, glycosylated hemoglobin (HbA1c), lipid profile, and serum creatinine. Blood pressure (BP) was measured on both arms at an interval of 1 min or more in a sitting position using a standard sphygmomanometer in a stable state after sufficient rest, and the higher of the two values was determined as BP. If systolic BP (SBP) or diastolic BP exceeded 140 or 90 mm Hg, respectively, the measurement was repeated after 5 min, and the average was recorded. All laboratory data were drawn on the exact same day as the brain MRI protocol, as part of the comprehensive routine health screening package.

### 2.3. cSVD Imaging Markers

Data from an MRI with a 1.5T or 3.0T scanner for cSVD markers including WMH, lacune (asymptomatic), CMB, and enlarged perivascular space (EPVS) were obtained. The basic slice thickness of the images was 5 mm in the axial plane. All of the obtained imaging data was rated by a stroke neurologist (J.-H.P.) blinded to the clinical information. The severity of WMH was rated, using the visual rating scale proposed by Fazekas scores ranging from 0 to 3 [9] on T2-FLAIR (fluid-attenuated inversion recovery) imaging. All included participants in this cohort exhibited completely symmetric bilateral WMH distribution, and no inter-hemispheric asymmetry was observed. Lacune was defined as 3 to 15 mm cerebrospinal-fluid-filled cavities showing hyperintensity on T2-weighted image and hyperintense rim on T2-FLAIR in the basal ganglia, internal capsule, corona radiata, centrum semiovale, or brainstem. CMB was defined as round hypointense lesions on T2-weighted gradient echo-images with a diameter ≤ 10 mm. EPVS was defined as round, oval, or linear-shaped lesions showing hyperintensity on T2-weighted images and hypointensity on FLAIR images without a hyperintense rim. EPVS was counted at the level of the basal ganglia and centrum semiovale on the slide with the highest number affected in one hemisphere and rated 0 (none), 1 (1–10), 2 (11–20), 3 (21–40) and 4 (>40) [10].

### 2.4. Total cSVD Score

The total cSVD score was calculated using a 5-point ordinal scale [11]. A point was awarded if WMH was found to be Fazekas score 2 or 3, one or more lacunes were present, one or more CMBs were present, or EPVS was found to be of rating score 2 or more (>10) [10]. Accordingly, the total score of cSVD ranged from 0 to 4.

### 2.5. Outcome Measures

The primary outcome for this analysis was the first occurrence of symptomatic stroke after the baseline health examination, including stroke of any type (ischemic or hemorrhagic stroke), ischemic stroke, and hemorrhagic stroke. These events were ascertained according to the International Classification of Disease, 10th Revision, Clinical Modification (ICD-10-CM) codes (I63 and I61) based on neuroimaging-confirmed clinical diagnosis. To ensure complete and rigorous capture of all clinical outcomes and prevent any missing events due to patients seeking treatment at external medical institutions, the long-term follow-up data of this single-center cohort were comprehensively tracked by cross-referencing and merging the dataset with the National Health Insurance Service (NHIS) claims database using unique de-identified personal identification numbers. In South Korea, the NHIS is a compulsory, single-payer national health insurance system that covers the entire population, meaning that any symptomatic stroke requiring hospitalization or medical care at any institution nationwide is mandatorily recorded under the specific ICD-10-CM codes. The ischemic stroke outcome encompasses all symptomatic events regardless of their etiology. The secondary outcome was all-cause death, which was confirmed by Statistics Korea’s data.

### 2.6. Statistical Analysis

Data were summarized as mean ± standard deviation (SD) or number of participants (percentage), as appropriate. Comparisons across the groups were examined using the chi-square test or Fisher’s exact test for categorical variables and Student’s *t* test for continuous variables. Participants were compared based on the use of antiplatelet agents. Baseline demographic and clinical covariates were preselected based on previous studies of factors that influence future stroke events. Participants receiving no antiplatelet drug were set as the reference group for the purposes of comparison. Comparisons across cSVD groups were examined using Pearson’s chi-square test for categorical variables and the one-way analysis of variance (ANOVA) for continuous variables. To adjust for potential confounders, covariates were selected based on baseline differences across the cSVD score groups (*p* < 0.05) and clinical relevance. Cox proportional hazard regression analyses were performed to estimate the risk of outcome events during the follow-up period after adjusting for baseline covariates including age, sex, hypertension, diabetes mellitus, smoking, educational age, SBP, regular exercise, antihypertensive agent, antidiabetic agent, and antiplatelet agent. Regardless of the total number of health examination visits per participant, only the data from their first (initial) check-up visit were utilized as baseline covariates in the Cox proportional hazards model for stroke outcomes and survival status. Subgroup comparisons of cSVD groups between scores 0 and 3–4 for the hazards of outcome events were performed with respect to participants’ demographics including age (<60 vs. ≥60 years); sex; presence of lacune (0 versus ≥1); WMH (Fazekas score 1 versus 2–3); EPVS (0–10 versus >10); and therapeutic medications including antiplatelet agents, antihypertensive agents, and lipid modifiers using Cox proportional hazards regression model. The subgroup comparisons were made by including appropriate interaction effects between the subgroups and cSVD scores in the multivariable model. The interaction between cSVD scores and variables for predicting the risk of vascular outcomes was assessed by including the appropriate interaction terms in the model. Results were expressed as hazard ratios (HRs) and 95% confidence intervals (CIs). Kaplan–Meier curves were fit by the log-rank tests with Dunnett–Hsu multiple comparisons for cSVD score. Additionally, a post hoc power analysis was performed to assess the statistical power for detecting differences in stroke outcomes between groups based on antiplatelet use, given the observed event rates. All statistics were performed based on STATA version 15.1 (StataCorp., College Station, TX, USA) and all reported two-tailed *p* values of <0.05 were considered statistically significant.

## 3. Results

### 3.1. Participant Characteristics

Of the 3703 participants, after excluding 2031 patients (54.8%) who had no available cSVD data, a total of 1672 participants were included in this study (Supplementary Figure S1). Of 1672 participants, 1002 were men (59.9%). The mean age was 57.1 ± 8.5 years, and the mean WMH Fazekas score was 1.2 ± 0.4 (median, 1; interquartile range [IQR], 1–1) and mean cSVD 0.7 ± 1.0 (median, 0; IQR, 0–1). The study participants had lacune in 23.7%, CMB in 11.4%, and EPVS (>10) in 15.5%. Of 1672 participants, 260 (15.6%) took antiplatelet agents.

### 3.2. Association of WMH with Outcome Events

**Figure 1:**
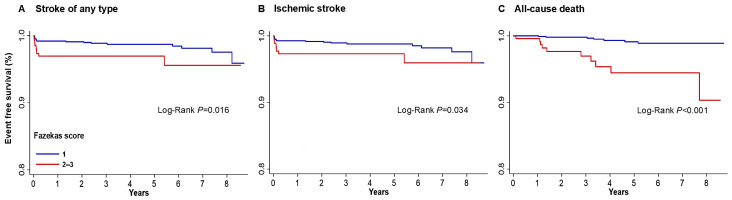
Kaplan–Meier curves for the outcome events of (**A**) stroke of any type, (**B**) ischemic stroke, and (**C**) all-cause death by WMH severity. WMH, white matter hyperintensities.

During the mean follow-up period of 3.5-years (median, 3.0; IQR, 1.2–5.9), a total of 28 (1.7%) strokes of any type, 26 (1.6%) ischemic strokes, 2 (0.2%) hemorrhagic strokes, and 17 (1.0%) all-cause deaths were observed. Supplementary Table S1 provides the baseline characteristics of study participants by WMH severity. Participants with Fazekas scores 2–3 were older, more likely to have higher levels of cSVD score, SBP, and HbA1c, greater frequencies of hypertension, diabetes, lacune, CMB, and EPVS (>10), and taken medications including antiplatelet, antihypertensive, and antidiabetic agents, and lipid modifier; however, frequencies of male sex, smoking and regular exercise, educational age, and levels of total cholesterol, low-density lipoprotein cholesterol, and triglycerides were lower than participants with Fazekas score 1. Supplementary Table S2 shows the unadjusted outcome events by WMH severity. Compared with participants with Fazekas score 1, participants with Fazekas scores 2–3 were significantly associated with higher risk of stroke of any type (HR, 2.56; 95% CI, 1.16–5.65), ischemic stroke (HR, 2.40; 95% CI, 1.04–5.51), and all-cause death (HR, 7.61; 95% CI, 2.89–19.99) but not of hemorrhagic stroke. Figure 1 depicts the Kaplan–Meier curves for the outcome events of (A) stroke of any type, (B) ischemic stroke, and (C) all-cause death by WMH severity during the mean 3.5-year follow-up period. A higher risk was seen in the group with Fazekas scores 2–3 (*p* = 0.016, *p* = 0.034, and *p* < 0.001, respectively, by log-rank test). Table 1 presents the adjusted results of association between WMH severity and outcome events showing loss of significance for stroke outcomes and all-cause death.

### 3.3. Association of cSVD with Outcome Events

Table 2 presents the baseline characteristics by cSVD score. Participants with higher cSVD score were older, more likely to have higher levels of SBP and HbA1c, have greater frequencies of hypertension, diabetes, and high SBP (≥140 mmHg), and take medications including antiplatelet, antihypertensive and antidiabetic agents; however, frequencies of smoking and regular exercise, and educational age were more likely to be lower in participants with higher cSVD scores.

**Figure 2:**
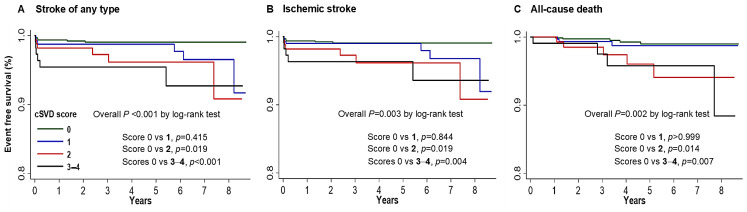
Kaplan–Meier curves for the outcome events of (**A**) stroke of any type, (**B**) ischemic stroke, and (**C**) all-cause death by cSVD score. cSVD, cerebral small-vessel disease.

Supplementary Table S3 demonstrates the unadjusted outcome events by cSVD score. Compared with score 0, a higher score was associated with greater risk of stroke of any type (HR, 4.35; 95% CI, 1.51–12.53 for score 2; and HR, 6.76; 95% CI, 2.34–19.49 for score 3–4); ischemic stroke (HR, 4.34, 95% CI, 1.51–12.52 for score 2; and HR, 5.61; 95% CI, 1.83–17.16 for scores 3–4); and all-cause death (HR, 5.62; 95% CI, 1.63–19.43 for score 2; and HR, 6.70; 95% CI, 1.80–24.95 for score 3–4). Figure 2 depicts the Kaplan–Meier curves for the outcome events of (A) stroke of any type, (B) ischemic stroke, and (C) all-cause death according to cSVD score during the mean 3.5-year follow-up period. A higher risk was seen in the group with cSVD scores 3–4 (*p* < 0.001, *p* = 0.003, and *p* = 0.002, respectively, by log-rank test). Compared with cSVD score 0, the risk of the three key outcome events was significantly higher in score 2 (*p* = 0.019, *p* = 0.019, and *p* = 0.014, respectively) and in scores 3–4 (*p* < 0.001, *p* = 0.004, and *p* = 0.007, respectively) by Dunnett–Hsu multiple comparison tests.

Table 3 presents the adjusted results of stepwise association between cSVD score and outcome events. Compared with cSVD score 0, scores 3–4 persisted as an independent predictor of stroke of any type (HR, 8.45; 1.53–46.53), but not of ischemic stroke or all-cause death. The adjusted HRs of covariates included in the Cox model appear in Supplementary Table S4. Among them, antiplatelet medication was not associated with less risk of stroke outcomes.

### 3.4. Subgroup Analysis

**Figure 3:**
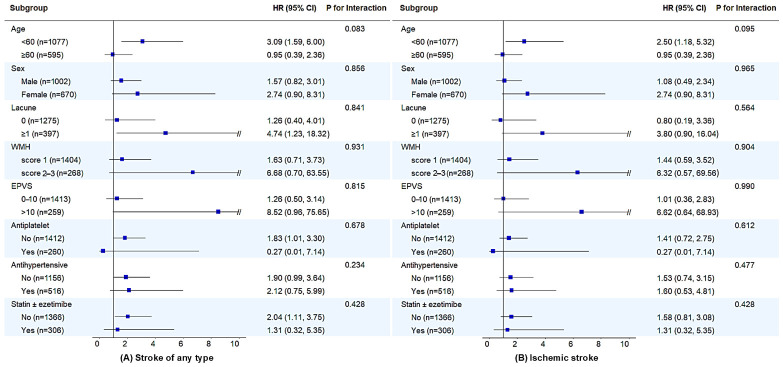
Subgroup analysis of stroke outcomes. WMH, white matter hyperintensities; EPVS, enlarged perivascular space; HR, hazard ratio; CI, confidence interval.

The interaction effect between subgroups and cSVD score on the risk of stroke of any type and ischemic stroke is provided in Figure 3. No significant interaction was noted between cSVD score severity and antiplatelet effect on stroke outcome events (*P*_interaction_ = 0.678 for stroke of any type, and *P*_interaction_ = 0.612 for ischemic stroke). When analyzing the direct effect of antiplatelet medication, the use of antiplatelet agents (*n* = 260) did not show a statistically significant reduction in the risk of stroke of any type (HR = 0.27; 95% CI: 0.01–7.14) or ischemic stroke (HR = 0.27; 95% CI: 0.01–7.14) compared to non-use (*n* = 1412). Likewise, the interactions between cSVD score and subgroups including age, sex, presence of lacune, WMH Fazekas scores 2–3 versus 1, EPVS (>10), and concomitant medications cSVD score were all non-significant.

### 3.5. Antiplatelet Agent and Outcome Events

Table 4 demonstrates the incidence rate and unadjusted outcome events, indicating that antiplatelet use was not significantly associated with a reduced risk of stroke of any type, ischemic stroke, hemorrhagic stroke, or all-cause death. A post hoc power analysis based on these observed rates revealed a statistical power of 0.12 for both stroke of any type and ischemic stroke, 0.00 for hemorrhagic stroke, and 0.03 for all-cause death at a two-sided alpha level of 0.05. These values reflect the limited number of outcome events within this relatively healthy, ASCVD-naïve cohort.

## 4. Discussion

In this retrospective analysis of a consecutive cohort for health check-up during the mean 3.5-year follow-up period, Kaplan–Meier curves showed that individuals with WMH Fazekas scores of 2–3 (versus 1) and those with cSVD scores of 2 and 3–4 (versus 0) were associated with increased risk of stroke of any type, ischemic stroke, and all-cause death among ASCVD-naïve adults. In the multivariable analysis, these associations were attenuated, with stroke of any type remained significant only in individuals with cSVD scores of 3–4. These findings suggest that WMH severity and cSVD scores have limited strength as independent predictors of stroke outcomes in an ASCVD-naïve population.

As shown in Supplementary Tables S1 and S2, participants with a Fazekas score of 2–3 or a higher cSVD score were older and more likely to have higher levels of SBP and HbA1c, along with greater frequencies of hypertension, diabetes, and individual cSVD components. While these medical conditions were associated with a higher frequency of related medications, the specific clinical rationale for antiplatelet prescriptions cannot be determined from our retrospective data. In these ASCVD-naïve individuals, antiplatelet therapy might have been initiated to manage the overall cardiovascular risk profile, reflecting real-world clinical practice where a high cSVD or WMH burden is often perceived as a marker of increased vascular risk. However, antiplatelet agents did not significantly reduce the risks of stroke outcomes and all-cause death, as shown in Table 4. Furthermore, the interactions between antiplatelet agent and cSVD score on stroke outcome events were not significant (Figure 3) and nor was the independent association of antiplatelet agent (Supplementary Table S4). The suspected reasons why antiplatelet agents showed a null effect in this ASCVD-naïve cohort, compared to stroke patients, include the participants’ relatively younger age, the lower frequency of risk factors, and the low number of outcome events. Furthermore, participants’ increased attention to their health following check-ups is also likely a contributing factor.

Taken together, our findings should be interpreted with caution. As the statistical power of 0.12 for stroke of any type indicates that the study was underpowered to confirm a definitive null effect, the lack of statistical significance should not be misinterpreted as definitive evidence of an absence of effect. Instead, it offers an exploratory view of real-world clinical practices where the preventive efficacy of antiplatelet therapy has not been robustly observed.

Our findings might support both AHA/ASA and ESO guideline recommendations that antiplatelet therapy should not be commenced for incidental WMH or cSVD in the absence of another indication due to the lack of high-quality evidence supporting efficacy [5,6]. As such, our study suggests that for incidental WMH or cSVD in ASCVD-naïve adults, it would be more pragmatic to prioritize the investigation and control of underlying risk factors before initiating antiplatelet therapy. This approach is in accordance with a consensus statement that recommends the initial identification and management of vascular risk factors in this population [5].

Hypertension is the most important contributor to the incidence and advancement of WMH or cSVD score [12]. In a recent Mendelian randomization study [13], elevated BP was significantly associated with higher WMH volume, an increased risk of EPVS, and lacunar stroke. A meta-analysis [14] and the SPRINT-MIND study (Systolic Blood Pressure Intervention Trial Memory and Cognition in Decreased Hypertension) [15] have demonstrated that intensive BP lowering can slow down and mitigate the progression of WMH. A recent INFINITY trial showed that 24 h mean systolic BP of ≤130 mm Hg versus ≤145 mm Hg effectively mitigates the worsening of WMH and concurrently leads to fewer cardiovascular complications [16]. Diabetes mellitus [17,18,19] and smoking [20,21,22] are shown to be associated with increased risk of WMH or cSVD burden. Yet, the evidence demonstrating a clinical benefit of tight glycemic control or smoking cessation on the progression of WMH or cSVD is currently lacking. A systematic review showed that statin can reduce the accrual of covert brain infarcts (Risk Ratio, 0.63: 95% CI, 0.46–0.88) [23]. Currently, there is no direct evidence for the beneficial effect of statin on high WMH or cSVD score per se, unless high ASCVD risk is expected.

While a detailed debate on the optimal targets for BP or glycemic control is beyond the scope of this study, our findings emphasize that clinical priority for ASCVD-naïve individuals with high cSVD scores should remain focused on the rigorous management of these underlying vascular risk factors. Given the limited evidence and low statistical power observed for the preventive effect of antiplatelets in our cohort, individualized risk factor modification appears to be a more grounded clinical strategy rather than the empirical prescription of antiplatelet agents.

### Limitations

There are several limitations to this study. First, the assignment of antiplatelet agents was not randomized but based on the physician’s discretion, which raises the potential for selection bias. From a cost perspective, it would not be practically easy to attempt such validation through randomized controlled trials targeting healthy adults. Nevertheless, it is crucial to note that antiplatelet agents were preferentially prescribed to individuals deemed to be in the high-risk group. Second, because this was a retrospective cohort study and the participants were restricted to those with a Fazekas score ≥1, more than half of the initial screening population was excluded. This selection process may have yielded potential selection bias, and the findings may not be generalizable to the entire healthy screening population. Third, this was a single-academic-center study examining a small cohort of Korean participants from a health check-up and the sample size of WMH Fazekas scores 2–3 and cSVD scores 3–4 was small, with confirmation of our findings needed in larger collaborations. Fourth, we did not have information on the individual subject’s BP trajectory and continuous control level of risk factors. Otherwise, it would have helped address the significance of risk factor control over WMH and cSVD scores. Furthermore, single-baseline neuroimaging assessment precluded the evaluation of trends over time in cSVD progression. Finally, our study population was fairly young (mean age 57.1) with a relatively low prevalence of vascular risk factors and individual cSVD components. There is a possibility that the results might be different in older participants who have greater burdens of vascular risk factors and cSVD scores.

## 5. Conclusions

Although our study may be underpowered to confirm a definitive null effect, the lack of a robust preventive effect suggests that empirical antiplatelet therapy should be approached with caution in ASCVD-naïve individuals with cSVD until larger prospective trials are available. Prioritizing the identification and modification of the risk factors underlying these neuroimaging findings may represent a more clinically pragmatic approach. Our preliminary findings await future validation through large-scale, prospective studies across more ethnically diverse cohorts.

## Figures and Tables

**Table 1:** Adjusted HR for outcome events according to WMH severity.

WMH	Stroke of Any Type *		Ischemic Stroke		All-Cause Death	
Fazekas score	HR (95% CI) ^†^	*p*	HR (95% CI) ^†^	*p*	HR (95% CI) ^†^	*p*
1 (*n* = 1404)	1 [Referent]		1 [Referent]		1 [Referent]	
2–3 (*n* = 268)	2.58 (0.71–9.31)	0.148	1.73 (0.41–7.25)	0.455	1.93 (0.41–9.21)	0.408

WMH, white matter hyperintensities; HR, hazard ratio; CI, confidence interval. * Ischemic or hemorrhagic stroke. ^†^ Adjusted for age, sex, hypertension, diabetes mellitus, smoking, educational age, cSVD score, systolic BP, regular exercise, LDL-C, antiplatelet agent, antihypertensive agent, antidiabetic agent, and lipid modifier.

**Table 2:** Baseline characteristics of study participants according to cSVD score.

	cSVD Score				
	0	1	2	3–4	*p* *
Number of participants	981	405	171	112	
Demographics					
Age, years	54.4 ± 7.5	58.6 ± 7.8	62.8 ± 7.8	66.7 ± 7.3	<0.001
Male sex	596 (60.8)	246 (60.7)	99 (57.9)	58 (51.8)	0.216
Medical history					
Hypertension	226 (23.0)	153 (37.8)	79 (46.2)	57 (50.9)	<0.001
Diabetes mellitus	100 (10.2)	59 (14.6)	26 (15.2)	18 (16.1)	0.039
Current smoking	175 (17.8)	70 (17.3)	24 (14.0)	9 (8.0)	0.002
Family history of stroke	146 (14.9)	67 (16.5)	28 (16.4)	19 (17.0)	0.511
Educational age, year	13.6 ± 3.1	12.8 ± 3.3	12.1 ± 3.7	10.7 ± 3.5	<0.001
Vital signs					
Systolic BP, mm Hg	122.5 ± 14.0	124.2 ± 14.1	128.7 ± 13.8	133.3 ± 17.7	<0.001
Systolic BP category					
<120 mm Hg	388 (39.6)	134 (33.1)	39 (22.8)	20 (17.9)	<0.001
120 to <140 mm Hg	466 (47.5)	210 (51.9)	93 (54.4)	51 (45.5)	
≥140 mm Hg	127 (13.0)	61 (15.1)	39 (22.8)	41 (36.6)	
Diastolic BP, mm Hg	87.1 ± 25.4	86.2 ± 25.3	88.5 ± 25.2	93.4 ± 28.3	0.060
Body mass index, kg/m^2^	24.3 ± 3.0	24.4 ± 2.9	24.6 ± 2.8	24.8 ± 3.4	0.401
Regular exercise ^†^	274 (27.9)	122 (30.1)	42 (24.6)	21 (18.8)	0.001
Laboratory findings					
Glucose, mg/dL	100.3 ± 26.2	100.9 ± 24.7	102.7 ± 24.0	100.4 ± 20.2	0.703
HbA1c, %	5.8 ± 0.9	5.9 ± 0.9	6.0 ± 0.8	6.0 ± 0.7	0.002
Total cholesterol, mg/dL	200.5 ± 38.1	199.0 ± 38.0	196.5 ± 39.2	192.2 ± 37.1	0.123
LDL-C, mg/dL	134.0 ± 35.8	132.0 ± 37.1	129.3 ± 36.5	127.9 ± 33.1	0.185
Triglycerides, mg/dL	123.0 ± 78.4	123.9 ± 79.8	121.3 ± 70.2	110.3 ± 57.0	0.389
HDL-C, mg/dL	57.8 ± 15.2	58.5 ± 15.6	56.7 ± 16.1	55.1 ± 12.7	0.165
Creatinine, mg/dL	0.89 ± 0.2	0.89 ± 0.2	0.88 ± 0.2	0.88 ± 0.3	0.824
Concomitant medication					
Antiplatelet agent	140 (14.3)	60 (14.8)	33 (19.3)	26 (23.2)	0.062
Antihypertensive agent	226 (23.0)	153 (37.8)	79 (46.2)	57 (50.9)	<0.001
Antidiabetic agent	100 (10.2)	59 (14.6)	26 (15.2)	18 (16.1)	0.039
Lipid modifier	167 (17.0)	80 (19.8)	32 (18.7)	26 (23.2)	0.417

Values provided are number (%), mean ± SD, or median (interquartile range). cSVD, cerebral small-vessel disease; BP, blood pressure; HbA1c, glycosylated hemoglobin; LDL-C, low-density lipoprotein cholesterol; HDL-C, high-density lipoprotein cholesterol; Lipid modifier, statin with/without ezetimibe. * By Pearson’s chi-square test for categorical variables and Student’s *t* test for continuous variables as appropriate. ^†^ Determined if participant implemented at least 150 min of moderate-intensity aerobic physical activity per week, or 75 min of vigorous-intensity aerobic physical activity per week.

**Table 3:** Adjusted HR for outcome events according to cSVD score.

cSVD Score	Stroke of Any Type *		Ischemic Stroke		All-Cause Death	
	HR (95% CI) ^†^	*p*	HR (95% CI) ^†^	*p*	HR (95% CI) ^†^	*p*
0 (*n* = 981)	1 [Referent]		1 [Referent]		1 [Referent]	
1 (*n* = 405)	2.97 (0.91–9.69)	0.072	2.13 (0.61–7.41)	0.233	0.64 (0.10–4.02)	0.631
2 (*n* = 171)	1.43 (0.16–12.77)	0.747	1.22 (0.14–11.07)	0.858	1.25 (0.20–7.63)	0.810
3–4 (*n* = 112)	8.45 (1.53–46.53)	0.014	3.98 (0.60–26.42)	0.153	1.84 (0.26–13.06)	0.544

cSVD, cerebral small-vessel disease; HR, hazard ratio; CI, confidence interval. * Ischemic or hemorrhagic stroke. ^†^ Adjusted for age, sex, hypertension, diabetes mellitus, smoking, educational age, systolic blood pressure, regular exercise, antiplatelet agent, antihypertensive agent, and antidiabetic agent.

**Table 4:** Incidence rate and unadjusted HR for outcome events based on the use of antiplatelet agents.

	No Antiplatelet Use	Antiplatelet Use	*p*
Stroke of any type ^†^, *n* *	26	2	
Incidence rate/100 person-year (95% CI)	0.52 (0.35–0.76)	0.25 (0.06–1.00)	
Crude HR (95% CI)	1 [Referent]	0.44 (0.11–1.87)	0.269
Ischemic stroke, *n* *	24	2	
Incidence rate/100 person-year (95% CI)	0.48 (0.32–0.71)	0.25 (0.06–1.00)	
Crude HR (95% CI)	1 [Referent]	0.48 (0.11–2.05)	0.324
Hemorrhagic stroke, *n* *	2	0	
Incidence rate/100 person-year (95% CI)	0.04 (0.01–0.16)	—	
Crude HR (95% CI)	1 [Referent]	—	—
All-cause death, *n* *	16	1	
Incidence rate/100 person-year (95% CI)	0.31 (0.19–0.51)	0.12 (0.02–0.88)	
Crude HR (95% CI)	1 [Referent]	0.41 (0.05–3.05)	0.380

HR, hazard ratio; CI, confidence interval. * Total number of events during the follow-up period. ^†^ Ischemic or hemorrhagic stroke.

## Data Availability

The anonymized data used in this study are available from the corresponding author (J.-H.P.) upon reasonable request.

## Supplementary Materials

The following supporting information can be downloaded at https://www.mdpi.com/article/10.3390/jcm15145704/s1, Figure S1: Flowchart of participant inclusion; Table S1: Baseline characteristics of participants by WMH severity; Table S2: Unadjusted HR for outcome events according to WMH severity; Table S3: Unadjusted HR for outcome events according to cSVD score; Table S4: Adjusted HR of covariates for stroke outcome events according to cSVD score.
