# Supplementary material for: Antiplatelet Therapy for Stroke Prevention in Atherosclerotic Cardiovascular Disease-Naïve People with Cerebral Small-Vessel Disease: A Retrospective Cohort Study
Source: J Clin Med. 2026 Jul 21;15(14):5704. doi: 10.3390/jcm15145704 (PMC13413284; doi:10.3390/jcm15145704)
Supplement: Supplementary file 1 [file jcm-15-05704-s001.zip › jcm-4434157-supplementary.pdf]

0

SUPPLEMENTARY MATERIALS

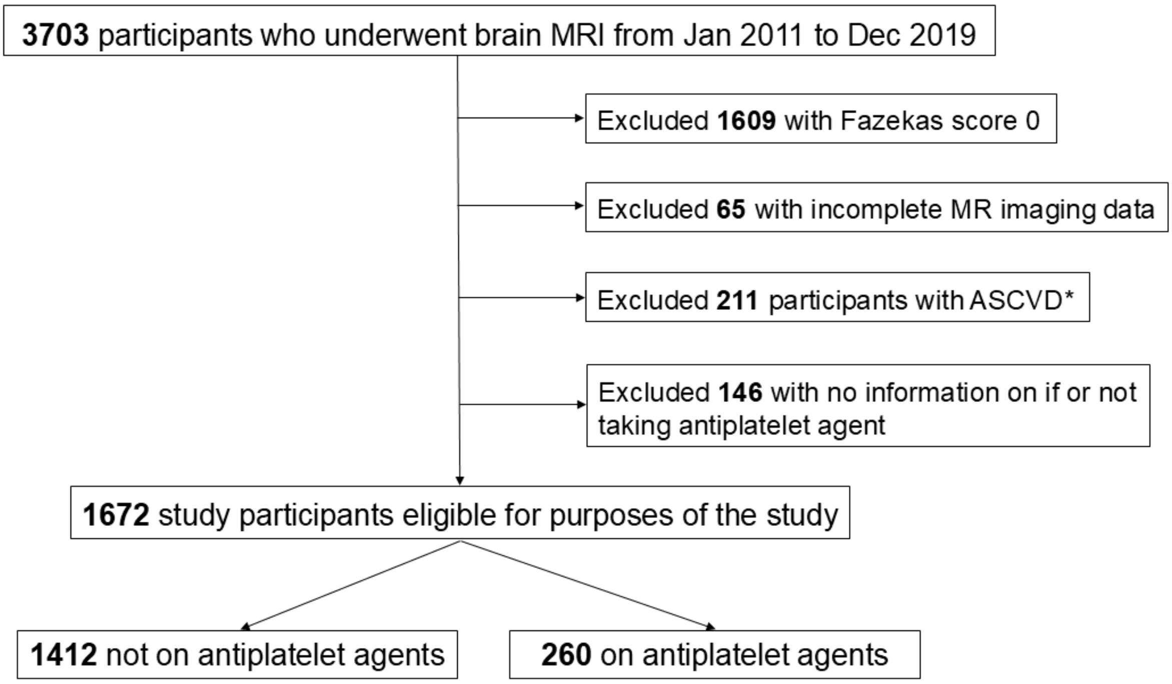

Supplementary Figure S1. Flowchart of participant inclusion. ASCVD, atherosclerotic cardiovascular disease. *Defined as a clinically diagnosed symptomatic stroke (either ischemic or hemorrhagic), myocardial infarction, angina pectoris, coronary revascularization, or peripheral arterial disease.

Supplementary Table S1. Baseline characteristics of participants by WMH severity.

	Fazekas score 1	Fazekas scores 2–3	p*
Number of participants	1404	268†	
Demographics			
Age, years	55.7 ± 7.7	64.6 ± 8.3	<0.001
Male sex	866 (61.7)	136 (50.8)	0.001
Medical history			
Hypertension	394 (28.1)	122 (45.5)	<0.001
Diabetes mellitus	152 (10.8)	52 (19.4)	<0.001
Current smoking	250 (17.8)	29 (10.8)	0.001
Family history of stroke	217 (15.5)	43 (16.0)	0.272
Educational age, year	12.4 ± 3.1	11.2 ± 3.6	<0.001
cSVD markers on MRI			
Lacune	0 (0, 4)	0.5 (0, 1)	<0.001
CMB	0 (0, 0)	0 (0, 1)	<0.001
EPVS (>10)	142 (10.1)	117 (43.7)	<0.001
cSVD score	0 (0, 1)	2 (1, 3)	<0.001
Vital signs			
Systolic BP, mm Hg	123.4 ± 14.2	128.6 ± 15.8	<0.001
Systolic BP category			<0.001
<120 mm Hg	513 (36.5)	68 (25.4)	
120 to <140 mm Hg	687 (48.9)	135 (50.4)	
≥140 mm Hg	204 (14.5)	65 (24.3)	
Diastolic BP, mm Hg	87.3 ± 25.5	88.3 ± 25.8	0.552
Body mass index, kg/m^2	24.4 ± 3.0	24.5 ± 3.1	0.464
Regular exercise‡	398 (28.4)	62 (23.11)	0.001
Laboratory findings			
Glucose, mg/dL	100.6 ± 25.6	101.3 ± 23.3	0.657
HbA1c, %	5.9 ± 0.9	6.1 ± 0.8	<0.001
Total cholesterol, mg/dL	200.3 ± .37.7	192.9 ± 39.6	0.003
LDL-C, mg/dL	133.7 ± 36.0	126.8 ± 35.9	0.004
Triglycerides, mg/dL	123.8 ± 78.2	113.4 ± 67.6	0.041
HDL-C, mg/dL	57.7 ± 15.4	58.0 ± 14.7	0.744
Creatinine, mg/dL	0.9 ± 0.2	0.9 ± 0.3	0.051
Concomitant medication			
Antiplatelet agent	203 (14.5)	57 (21.3)	0.005
Antihypertensive agent	394 (28.1)	122 (45.2)	<0.001
Antidiabetic agent	152 (10.8)	52 (19.4)	<0.001

Lipid modifier	240 (17.1)	66 (24.6)	0.003

Values provided are number (%), mean ± SD, or median (interquartile range). WMH, White matter hyperintensities; cSVD, cerebral small-vessel disease; CMB, cerebral microbleed; EPVS, enlarged perivascular space; BP, blood pressure; HbA1c, glycosylated hemoglobin; LDL-C, low-density lipoprotein cholesterol; HDL-C, high-density lipoprotein cholesterol; Lipid modifier, statin with/without ezetimibe. *By Pearson's Chi-square test for categorical variables and Student *t* test for continuous variables as appropriate. [†]n=254 for score 2; n=14 for score 3. [‡]Determined if implemented at least 150 minutes of moderate-intensity aerobic physical activity per week, or 75 minutes of vigorous-intensity aerobic physical activity per week.

Supplementary Table S2. Unadjusted HR for outcome events according to WMH severity.

WMH	Stroke of any type*		Ischemic stroke		Hemorrhagic stroke		All-cause death	
Fazekas score	HR (95% CI)	*p*	HR (95% CI)	*p*	HR (95% CI)	*p*		*p*
1 (n=1404)	1 [Referent]		1 [Referent]		1 [Referent]		1 [Referent]	
2–3 (n=268)†	2.56 (1.16–5.65)	0.020	2.40 (1.04–5.51)	0.040	5.25 (0.33–83.99)	0.241	7.61 (2.89–19.99)	<0.001

WMH, White matter hyperintensities; HR, hazard ratio; CI, confidence interval. *Ischemic or hemorrhagic stroke. †n=254 for score 2; n=14 for score 3.

Supplementary Table S3. Unadjusted HR for outcome events according to cSVD score.

cSVD score	Stroke of any type*		Ischemic stroke		All-cause death	
	HR (95% CI)	*p*	HR (95% CI)	*p*	HR (95% CI)	*p*
0 (n=981)	1 [Referent]		1 [Referent]		1 [Referent]	
1 (n=405)	2.44 (0.91–6.50)	0.075	2.13 (0.77–5.89)	0.143	1.52 (0.36–6.37)	0.566
2 (n=171)	4.35 (1.51–12.53)	0.007	4.34 (1.51–12.52)	0.007	5.62 (1.63–19.43)	0.006
3–4 (n=112)	6.76 (2.34–19.49)	<0.001	5.61 (1.83–17.16)	0.003	6.70 (1.80–24.95)	0.005

cSVD, cerebral small-vessel disease; HR, hazard ratio; CI, confidence interval. *Ischemic or hemorrhagic stroke.

Supplementary Table S4. Adjusted HR of covariates for stroke outcome events according to cSVD score.

Covariates	Stroke of any type*		Ischemic stroke	
	Adjusted HR (95%, CI)	*p*	Adjusted HR (95%, CI)	*p*
Age (1-yr difference)	0.99 (0.92−1.06)	0.773	1.02 (0.95–1.10)	0.576
Male sex	1.02 (0.30−3.43)	0.980	0.75 (0.21–2.73)	0.662
Hypertension	1.32 (0.44−3.96)	0.624	1.18 (0.36–3.88)	0.780
Diabetes mellitus	—†	—	—†	—
Smoking	0.88 (0.22–3.45)	0.850	1.23 (0.30−5.13)	0.772
Educational age	1.10 (0.92−1.31)	0.304	1.09 (0.90−1.31)	0.371
Systolic BP	1.01 (0.97−1.04)	0.706	1.01 (0.97−1.04)	0.796
Regular exercise	1.02 (0.35−3.02)	0.964	1.23 (0.40−3.76)	0.716
Antiplatelet use	0.80 (0.17–3.71)	0.772	0.89 (0.19−4.24)	0.881
Antihypertensive use	—†	—	—†	—
Antidiabetic use	—†	—	—†	—

cSVD, cerebral small-vessel disease; BP, blood pressure; HR, hazard ratio; CI, confidence interval. *Ischemic or hemorrhagic stroke. †Not computable due to too small sample size.
